# Synthesis and Discovery of Schiff Base Bearing Furopyrimidinone for Selective Recognition of Zn^2+^ and its Applications in Cell Imaging and Detection of Cu^2+^

**DOI:** 10.3389/fchem.2021.774090

**Published:** 2021-11-29

**Authors:** Yanggen Hu, Chao Luo, Lili Gui, Jing Lu, Juncai Fu, Xinya Han, Junkai Ma, Lun Luo

**Affiliations:** ^1^ Hubei Key Laboratory of Wudang Local Chinese Medicine, School of Pharmaceutical Sciences, Hubei University of Medicine, Shiyan, China; ^2^ Institute of Biomedicine, Hubei University of Medicine, Shiyan, China; ^3^ The First Clinical College, Hubei University of Medicine, Shiyan, China; ^4^ Department of Chemical Biology and Pharmaceutical Engineering, School of Chemistry and Chemical Engineering, Anhui University of Technology, Shiyan, China

**Keywords:** furo[2, 3-d]pyrimidinone, schiff base, fluorescence, Zn^2+^ recognition, Cu^2+^/Fe^2+^ detection, live cells imaging

## Abstract

A simplefuro [2,3-d]pyrimidinone-based Schiff base FPS was synthesized via aza-Wittig reaction and structure elucidation was carried out by spectroscopic studies FT-IR, 1H NMR, and 13C NMR and mass spectrometry. FPS showed weak fluorescence emission in methanol and the selectivity of FPS to different metal ions (Mn^2+^, Ca^2+^, Fe^2+^, Fe^3+^, Mg^2+^, Al^3+^, Ba^2+^, Ag^+^, Co^2+^, Na^+^, K^+^, Cu^2+^, Zn^2+^, Pb^2+^, Bi^3+^) were studied by absorption and fluorescence titration. The results show that FPS has selective fluorescence sensing behavior for Zn^2+^ ions and the limit of detection (LOD) was calculated to be 1.19 × 10^–8^ mol/L. Moreover, FPS-Zn^2+^ acts as a metal based highly selective and sensitive new chemosensor for Cu^2+^ ions and the LOD was calculated to be 2.25 × 10^–7^ mol/L. In accordance with the results and theoretical calculations, we suspected that the binding mechanisms of FPS to Zn^2+^ and Cu^2+^ were assigned to be the cooperative interaction of Zn^2+^(Cu^2+^)-N.

## Introduction

Metal ions have pivotal functions for the growth and development process of organisms, and it is of great significance to identify and monitor metal ions in the environment and in organisms (Domingo 1994; Schmidt et al., 2009; Garza-Lombo et al., 2018). Fluorescence analysis technology has received increased attention in view of its utility for selective recognition of metal ion owing to its high selectivity, low toxicity, real-time detection, convenient and simple operation, and relatively friendly environment (Ma et al., 2015; Chae et al., 2020; Nsanzamahoro et al., 2020). In the past few years, many researchers have been devoted to finding and developing of some site-specific small-molecule fluorescence probes for highly selective recognition of metal ions (Liang et al., 2017; Bae et al., 2018; Yang et al., 2019) and for analyzing different metal ions, which are widely distributed in organisms and environments worldwide (Wang et al., 2019; Kim et al., 2021; Klenner et al., 2021; Sannigrahi et al., 2021). Many diseases, such as Alzheimer’s disease (Isaev et al., 2020), neuron disease (Marchetti 2014), Parkinson’s disease (Park et al., 2015), ischemia (Yin et al., 2019), epilepsy, and certain types of cancer and so on (Hildebrand et al., 2015; Hershfinkel 2018; Wang et al., 2020), are caused by the excessive or insufficient intake of Zn^2+^ and Cu^2+^ ions.

**SCHEME 1:**
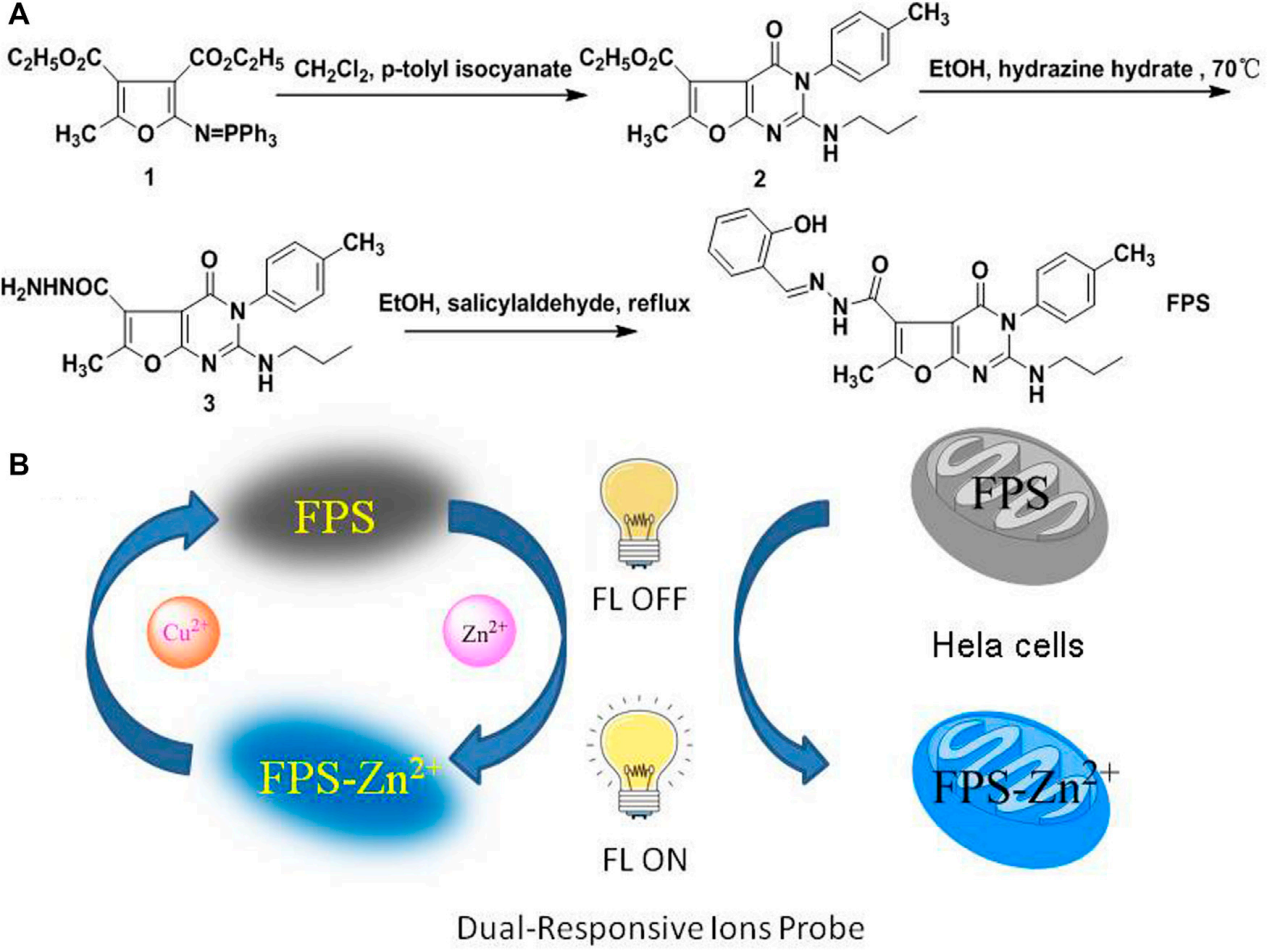
**(A)** The route of synthesis for **FPS**, **(B)** Dual-Response of **FPS** to Zn^2+^ and Cu^2+^, schematic of FPS applicated in cell imaging and detection Cu^2+^ ions.

Recently, we have focused on the synthesis of nitrogenous heterocyclic compounds via aza-Wittig reaction, attempting to apply and evaluate their biological activities (Hu et al., 2010; Hu et al., 2014; Wang et al., 2016a; Wang et al., 2016b; Li et al., 2016; Liu M.-G. et al., 2019; Liu N. et al., 2019; Gao et al., 2019; Liu et al., 2021). Herein, we designed a Schiff base bearing furopyrimidinone scaffold (**FPS**) synthesized from 2-hydroxy-benzaldehyde with furo [2,3-d]-pyrimidine-5-carbohydrazide (Scheme 1), and fluorescence analysis showed that **FPS** displayed highly selective recognition Zn^2+^ with no apparent interference from other metal ions in MeOH solution and its applications in cell imaging and detection Cu^2+^ ions.

## Experimental Sections

### Materials and General Methods

Unless otherwise stated, starting materials were commercially available and analytically pure, and the solvent was dried before use. The water used was redistilled water. The UV absorption and fluorescence emission spectra were recorded on a U-2550 Double-beam UV-Vis spectrophotometer (Japan) and a F-7000 fluorescence spectrometer (Japan), respectively. Melting points were recorded using an uncorrected X-4digital melting point apparatus. NMR were recorded on a Bruker Avance 400 MHz spectrometer (CDCl_3_ and DMSO-*d*_*6*_) with resonances relative to tetramethyl-silane (TMS) as an internal standard. Mass spectra (ESI) were recorded on a Waters XEVO G2-XSmass spectrometer. Fluorescence images of cells were analyzed by Dual photon confocal microscope (Olympus FV1000 MPE).

### Synthesis

#### Synthesis of 2-Ethyl-3,4-dihydro-6-methyl-4-oxo-2-(propylamino)-3-*p*-tolyl-furo[2,3-d]pyrimidine-5-carboxylate 3

Preparation of ethyl-3,4-dihydro-6-methyl-4-oxo-2-(propylamino)-3-*p*-tolyl-furo-[2,3-d]pyrimidine-5-carboxylate 2. As described in methods previously (Hu et al., 2012). A mixture of **1** (2.5 g, 5 mmol) and *p*-tolyl isocyanate (5 mmol) in anhydrous methylene dichloride 24 h at 0–5°C under N_2_, and then n-propylamine (5.2 mmol) was added, after the mixture was stirred for 1 h at room temperature. The solution was removed under reduced pressure and anhydrous EtOH (10 ml) with five drops of EtONa (10%) in EtOH was added. The mixture was stirred for 4 h at room temperature. The precipitated solid was collected and washed with ethanol to give ethyl 3,4-dihydro-6-methyl-4-oxo-2-(propyl-amino)-3-*p*-tolyl-furo [2,3-d]pyrimidine-5-carboxylate 2, which was used directly without further purification. A solution of **2** (5 mmol) and hydrazine hydrate (1 ml, 80%) in EtOH were stirred at 60–65°C for 15 h, after the solution was concentrated under reduced pressure and the residue recrystallized from CH_2_Cl_2_/EtOH (v:v = 4:1, 20 ml) to give 3,4-dihydro-6-methyl-4-oxo-2-(propylamino)-3-*p*-tolyl-furo [2,3-d]pyrimidine-5-carbohydrazide **3**, white solid, m. p.: 260–262°C; ^1^H NMR (400 MHz, DMSO-*d*_*6*_) δ: 0.8 (t, *J* = 8.0 Hz, 3H, CH_3_), 1.46–1.51 (m, 2H, CH_2_), 2.43 (s, 3H, CH_3_), 2.65 (s, 3H, CH_3_), 3.16–3.2 (m, 2H, NCH_2_), 4.44 (s, 2H, NH_2_), 6.45 (s, 1H, NH), 7.23–7.42 (m, 4H, ArH), 10.88 (s, 1H, NH); ^13^C NMR (100 MHz, DMSO-*d*_*6*_) *δ*: 165.45, 161.63, 160.70, 153.22, 153.00, 139.52, 132.11, 131.25, 129.24, 110.52, 93.38, 43.52, 22.11, 21.36, 13.48, 11.58; MS (70 eV) m/z (100%): Anal. calcd for C_18_H_21_N_5_O_3_ (M, 355.16), found [M + H^+^, 356.17].

#### Synthesis of N'-(2-hydroxybenzylidene)-3,4-dihydro-6-methyl-4-Oxo-2-(propylamino)-3-P-tolylfuro[2,3-d]pyrimidine-5-carbohydrazide FPS

A mixture of 3 (1.1 g, 3 mmol) and salicylaldehyde (3 mmol) in 25 ml ethanol was stirred for 10 h at 70–75°C, after the solution was concentrated under reduced pressure and the solid was collected and recrystallized from CH_2_Cl_2_/EtOH to give FPS (1.1 g, 84%). m. p.: 216–218°C. ^1^H NMR (400 MHz, DMSO-*d*_*6*_): δ = 13.55 (bs, 1H, ArOH), 8.35 (s, 1H, N = CH), 7.58–6.89 (m, 8H, Ar-H), 6.51 (bs, 1H, NH), 3.23 (q, *J* = 8.0, 2H, CH_2_), 2.72 (s, 3H, CH_3_), 2.45 (s, 3H, CH_3_), 0.82 (t, *J* = 8.0, 3H, CH_3_). ^13^C NMR (100 MHz, DMSO-*d*_*6*_): δ = 11.1, 13.3, 20.9, 21.6, 43.1, 92.6, 109.8, 116.3, 118.6, 119.3, 128.8, 129.7, 131.0, 131.4, 131.5, 139.3, 147.4, 152.9, 155.2, 157.4, 158.0, 160.7, 165.1. HRESI-MS m/z anal. calcd for C_25_H_25_N_5_O_4_ (M, 459.1985), found [M + H^+^, 460.1982].

### Spectroscopic Study

**FPS** was formulated into 1.0 mmol/L solution in DMSO and then diluted to definite concentration with methanol before the spectral experiment. The salts used in standard stock solutions of metal ions were MnSO_4_, Ca(NO_3_)_2_, CuSO_4_, Al(NO_3_)_3_, Ba(NO_3_)_2_, AgNO_3_, CoCl_2_, ZnSO_4_, FeSO_4_, NaCl, KCl, Pb(NO_3_)_2_, MgSO_4_, Fe(NH_4_) (SO_4_)_2_ in distilled water to prepare 0.050 mol/L. The spectral changes of the mixed solutions of **FPS** with various metal ions were studied by UV-Vis absorption and fluorescence spectroscopy at room temperature. The fluorescence emission of **FPS** were recorded with excitation at 480 nm.

### CCK8 Assay

The cytotoxicity of **FPS** was researched by CCK8 assay according to reported methods (Hou et al., 2019). Hela cells were cultured in DMEM medium containing 10% fetal bovine serum cell culture medium with 5% CO_2_ atmosphere at 37°C. The cells were transferred into 24-well plates and incubated for 24 h at 37°C. **FPS**, diluted to the desired concentrations (50–1,000 μmol/L) in culture medium, was added to the well. Then the original medium was removed after 24 h, and 10 μl of CCK-8 solution (5 mg/ml stock) was added to the Well and incubated for 2 h at 37°C. Absorbance at 450 nm was recorded with an enzyme-linked immunosorbent assay (ELISA) reader (Bio-Tek). The results showed that **FPS** exhibited low cytotoxicity against Hela cell lines with IC_50_ more than 500 μmol/L.

## Result and Discussion

### Fluorescence and UV-Vis Properties of FPS

**FIGURE 1:**
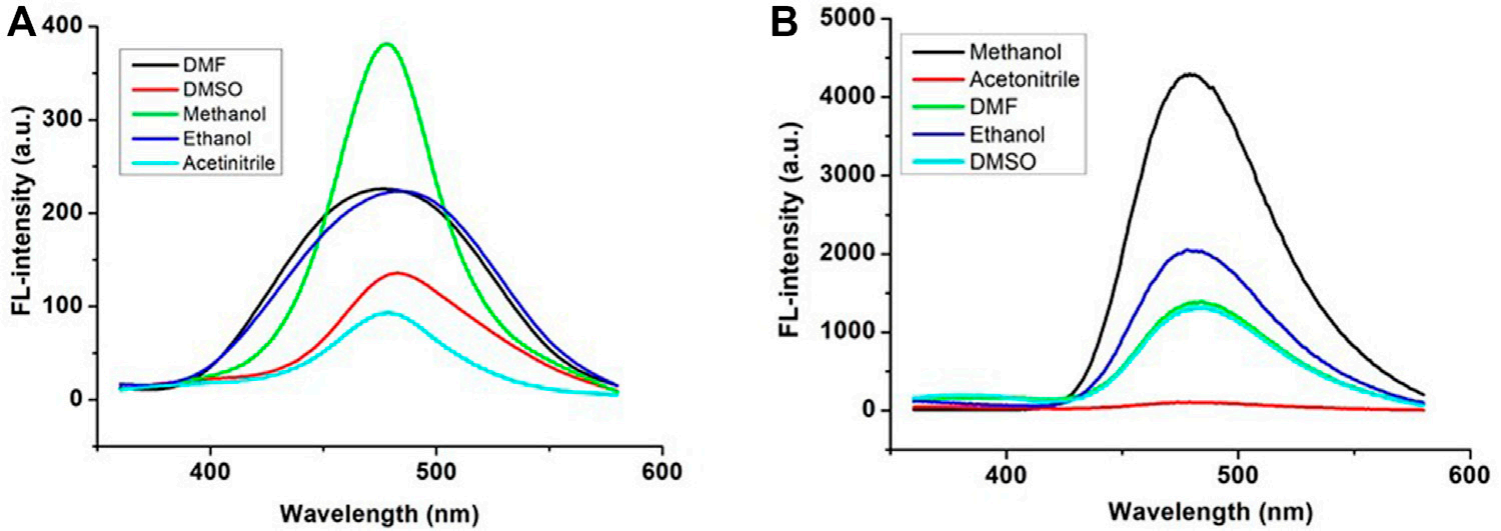
Fluorescence spectra of FPS **(A)** and FPS-Zn^2+^ **(B)** in different solvents.

**FPS** showed low fluorescence emission could be because of the photoinduced electron transfer (PET) process between the imine group and the benzene ring. To research the diverse solvent effect on **FPS** and **FPS-Zn** ^**2+**^, the emission and excitation spectra of **FPS** and **FPS-Zn** ^**2+**^ were recorded in different solvents [MeOH, EtOH, DMSO, and DMF (Figure 1)], respectively. These results showed that **FPS** and **FPS-Zn** ^**2+**^have higher fluorescence enhancement in MeOH than the other solvents.

### Selectivity of FPS

**FIGURE 2:**
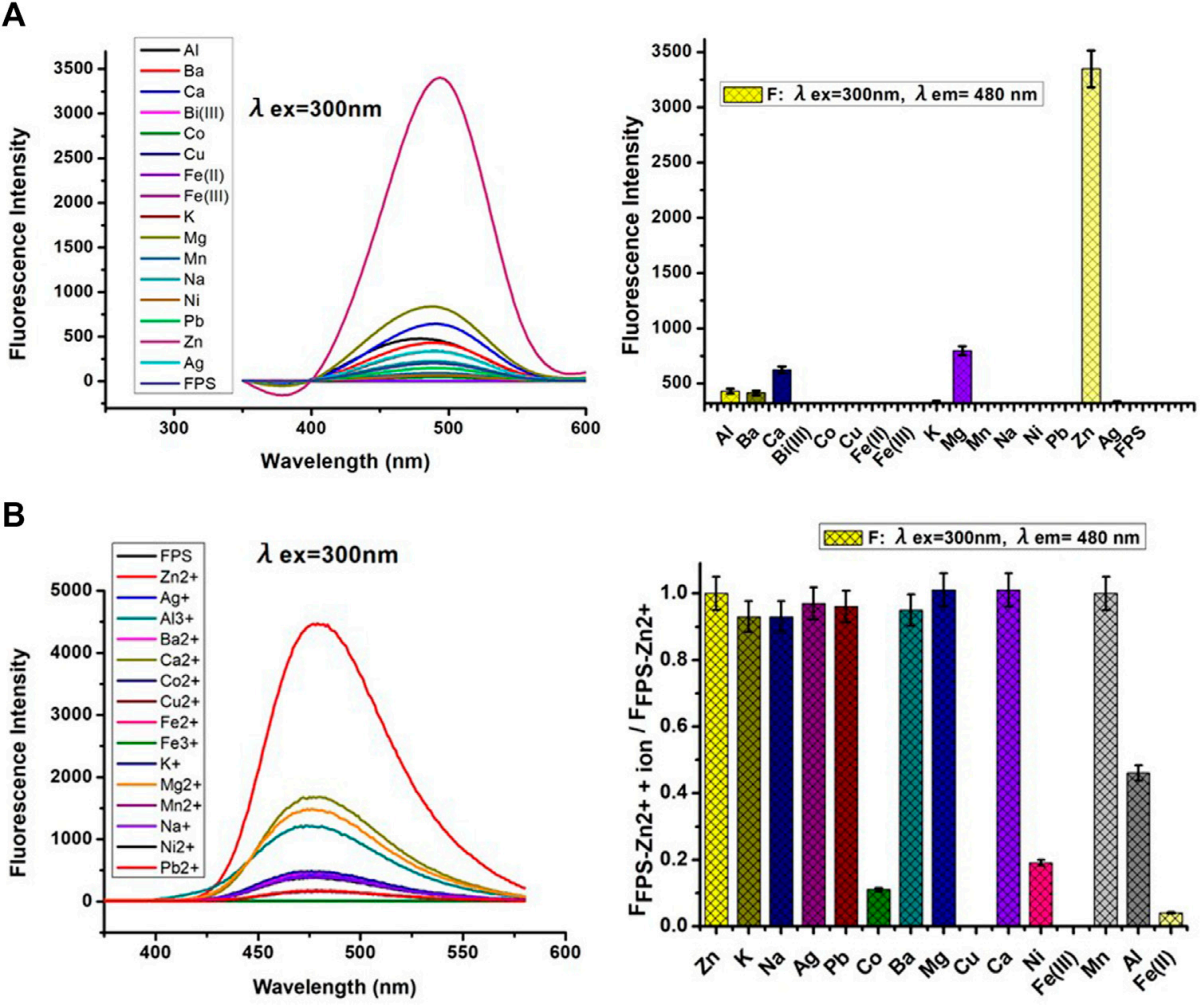
**(A)** Left: Fluorescent spectra of FPS (5 μmol/L) with various metal ions (10.0 equiv.) in methanol; Right: Dense bars indicate the fluorescence intensity (λ_ex_ = 300nm, λ_em_ = 480 nm) **(B)** Left: Fluorescent spectra of FPS (5 μmol/L) + 10.0 equiv. Zn^2+^ with various metal ions (10.0 equiv.) in methanol; Right: Dense bar portion indicates the fluorescence intensity (λ_ex_ = 300 nm, λ_em_ = 480 nm) in methanol solution, respectively.

The fluorescence properties of **FPS** with various metal ions (10.0 equiv. of Na^+^, K^+^, Ba^2+^, Zn^2+^, Mn^2+^, Mg^2+^, Ca^2+^, Ag^+^, Co^2+^, Ni^+^, Fe^3+^, Cd^2+^, Pb^2+^, Cu^2+^, Al^3+^) were investigated, respectively. Results showed that Zn^2+^ caused significant fluorescent enhancement at 480 nm with the color change from colorless to the light blue. These phenomena indicated that **FPS** could be used as fluorescent sensors for Zn^2+^ recognition (Figure 2A). The ability was also explored of **FPS** to detect Zn^2+^ in the presence of other metal ions. A competitive test was carried out, in which 10 equivalent other metal ions were added to the solution of **FPS** and Zn^2+^ ion (Figure 2B), respectively. Figure 2B shows that the fluorescence quenching of **FPS**-Zn^2+^ undergoes a significant change with the addition of Cu^2+^ and Fe^2+^, and there is a little interference with the addition of Al^3+^, Fe^3+^, Co^2+^ and Ni^2+^ ions.

### Job’s Plot Measurements

**FIGURE 3:**
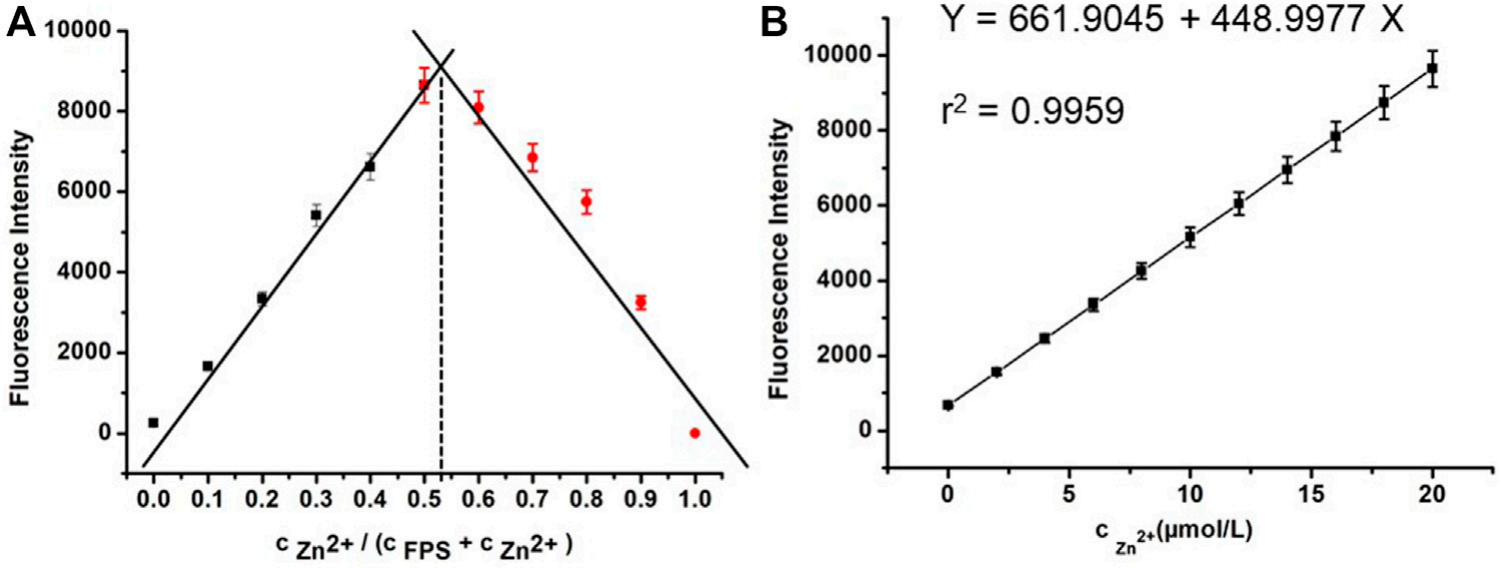
**(A)** Job’s plot for the stoichiometry determination of FPS and Zn^2+^ in the complexation and the fluorescence was performed as a function of the molar ratio [Zn^2+^]/([Zn^2+^] + [FPS]) **(B)** The fluorescence characteristics of FPS (20.0 μmol/L) with gradual addition of 0–20 μmol/L Zn^2+^ (λ_ex_ = 300 nm, λ_em_ = 480 nm).

**SCHEME 2:**
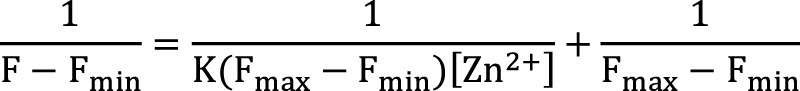
Benesi-Hildebrand equation. Where F_max_ and F_min_ are the fluorescence intensity of FPS in the presence and absence of zinc ions, respectively. F represents fluorescent intensities (at 480 nm) of FPS as a function of Zn^2+^ concentration. [FPS] = 20.0 μmol/L and [Zn^2+^] = 0–20 μmol/L.

Figure 3A is the Job’s plot of the fluorescence signal for FPS and Zn^2+^ solutions. The binding stoichiometry can be obtained from the plot. It revealed that a **1:1** binding was obtained between FPS and Zn^2+^ in methanol solution. Then the fluorescence characteristics of FPS to Zn^2+^ were further studied by fluorescence titration experiments (Figure 3B). The stability constant of FPS and Zn^2+^ was calculated to be 4.45×10^4^ (*r* ^2^ = 0.9626) from the nonlinear least squares fitting of the data, according to the Benesi-Hildebrand equation (Scheme 2). As shown in Figure 3B, with gradual addition of 0–20 μmol/L Zn^2+^ into the methanol solution of FPS (20.0 μmol/L), the fluorescence emission at 480 nm was increased gradually. Moreover, the detection limit of FPS to Zn^2+^ was calculated (LOD = 3σ/slope) to be 1.19 × 10^–8^ mol/L (*r* ^2^ = 0.9959).

### Concentration Effect of Cu^2+^and Fe^2+^ on Complex FPS-Zn^2+^

**FIGURE 4:**
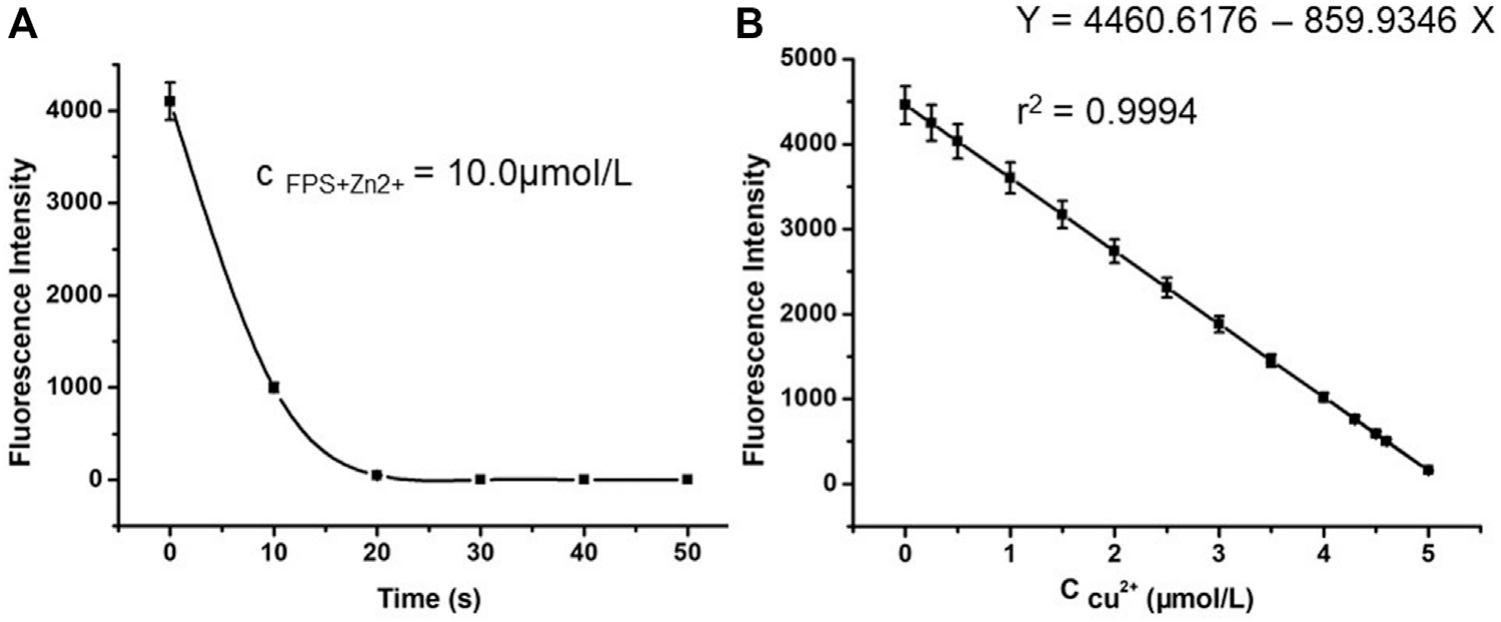
**(A)** The relationship between fluorescence intensity of 10.0 μmol/L FPS and the time **(B)** Fluorescence spectra (λ_ex_ = 300 nm) of 10.0 μmol/L FPS and 10 μmol/L Zn^2+^ in the presence of Cu^2+^ ion with various concentrations (from 0 to 5.0 μmol/L) in methanol solution.

Based on the results in Figure 2B, to evaluate further the effect of Cu^2+^ and Fe^2+^ concentration on the probe FPS for recognition Zn^2+^, respectively, the fluorescence properties of complex **FPS**-Zn^2+^ were studied in methanol solution (Figure 4). For Cu^2+^ ion, at low concentrations (c_Cu2+_ ≤ 5 μmol/L), the fluorescence emission was continuously quenched with the increase of Cu^2+^ and there was a good linear relationship. However, for Fe^2+^, there were no similar phenomena, the fluorescence emission at 480 nm was quenched completely, at that moment, with the addition of Fe^2+^ even in small amounts. Then to investigate the time-dependent of fluorescence quenching for Cu^2+^, as shown in Figure 4, the florescence intensity tended to be stable after adding the Cu^2+^ ion 15 seconds. In addition, the detection limit of complex **FPS** **-**Zn^2+^ to Cu^2+^ was calculated (LOD = 3σ/slope) to be 2.25 × 10^−7 ^mol/L (*r* ^2^ = 0.9987). These results show that a fluorescent probe composed of complex **FPS** **-**Zn^2+^ could be used as fluorescent sensors for Cu^2+^ detection real-time and for Fe^2+^ qualitative determination.

### pH Tolerance of Complex FPS-Zn^2+^

**FIGURE 5:**
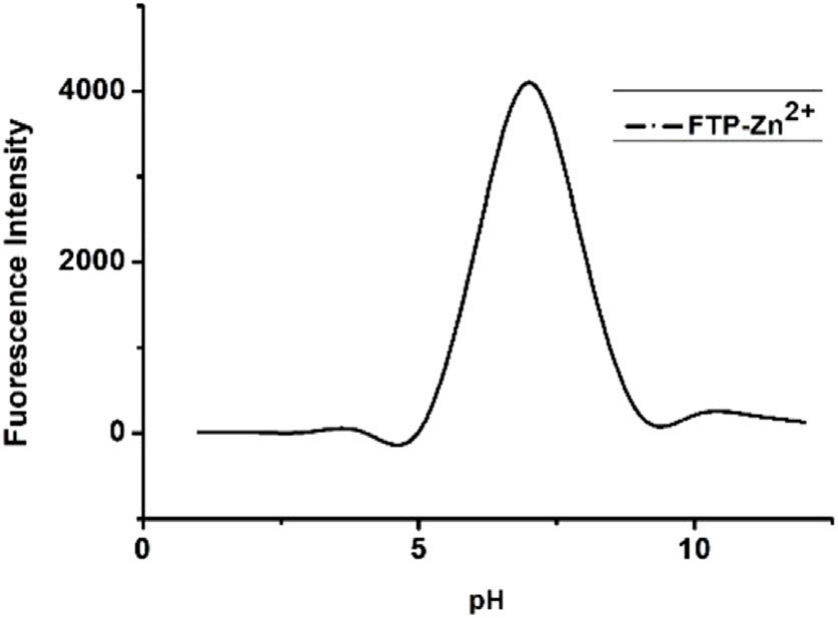
Fluorescent spectra of FPS (5 μmol/L) + 1.0 equiv. Zn^2+^ with pH (1–12) in DMSO/H_2_O.

As shown in Figure 5 **,** the fluorescent spectra of FPS (5 μmol/L) + 1.0 equiv. Zn^2+^ with pH (1–12) in DMSO/H_2_O be studied, in the neutral solution, the fluorescence intensity of FPS + Zn^2+^ complex is strongest. At the same time, when the pH lower than five or higher than 10, the fluorescent of FPS + Zn^2+^ complex is quenching.

### ^1^H NMR Experiment

**FIGURE 6:**
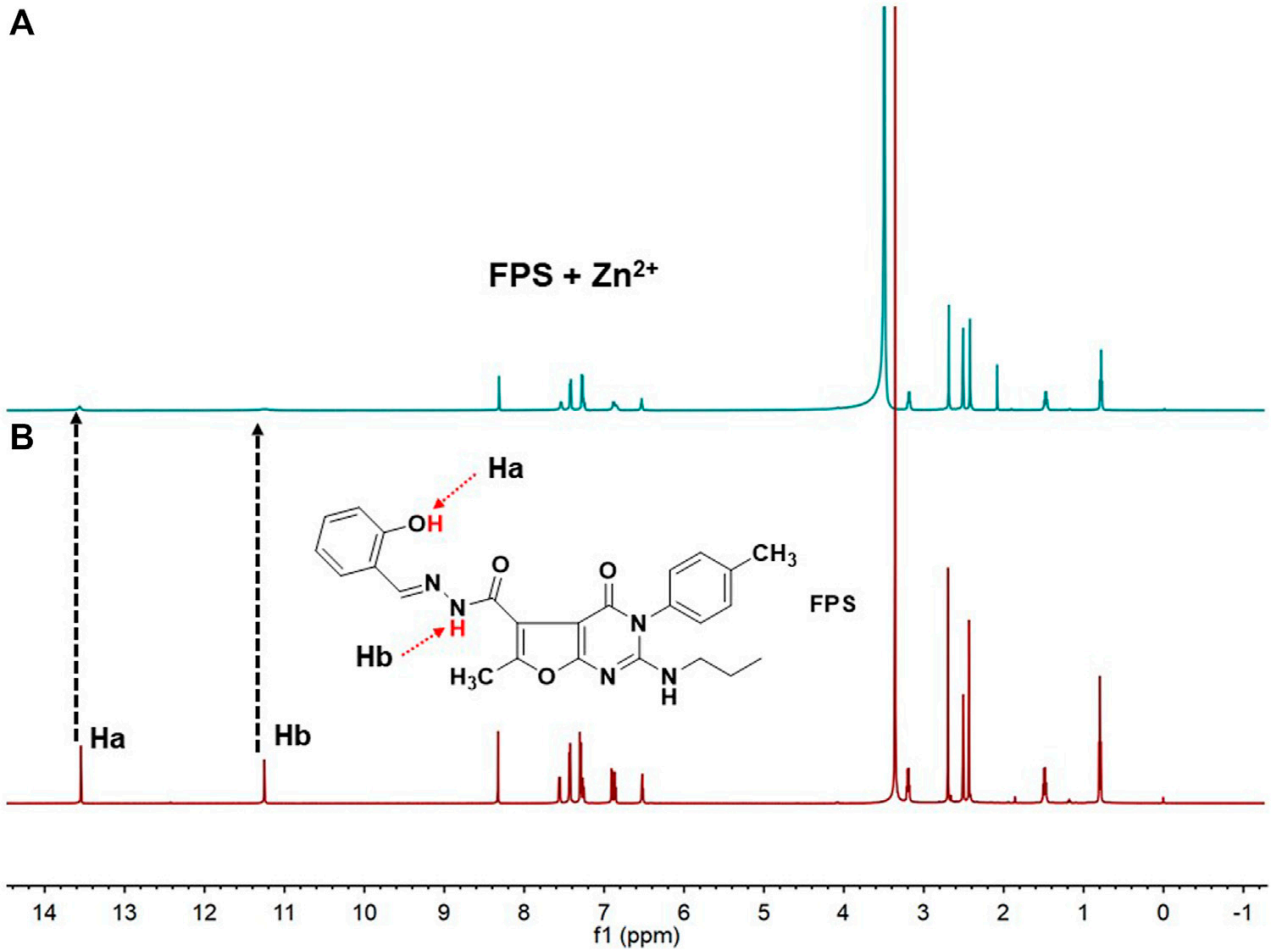
^1^H NMR spectra (400 MHz, DMSO-*d6*, 298 K) of **(A)** FPS (6 mm) + Zn^2+^ (6 mm) **(B)** free FPS (6 mm). This indicated that phenolic hydroxyl and amide play important roles of FPS-Zn^2+^ complex.

The binding ability of **FPS** with Zn^2+^ was evaluated using ^1^H NMR. As shown in Figure 6, when Zn^2+^ was added to **FPS**, the protons on the phenolic hydroxyl and amide of the **FPS** Ha and Hb were nearly despaired, respectively. This indicated that phenolic hydroxyl and amide coordinated to Zn^2+^ and the **FPS**-Zn^2+^ complex was formed.

### Theoretical Calculations

**FIGURE 7:**
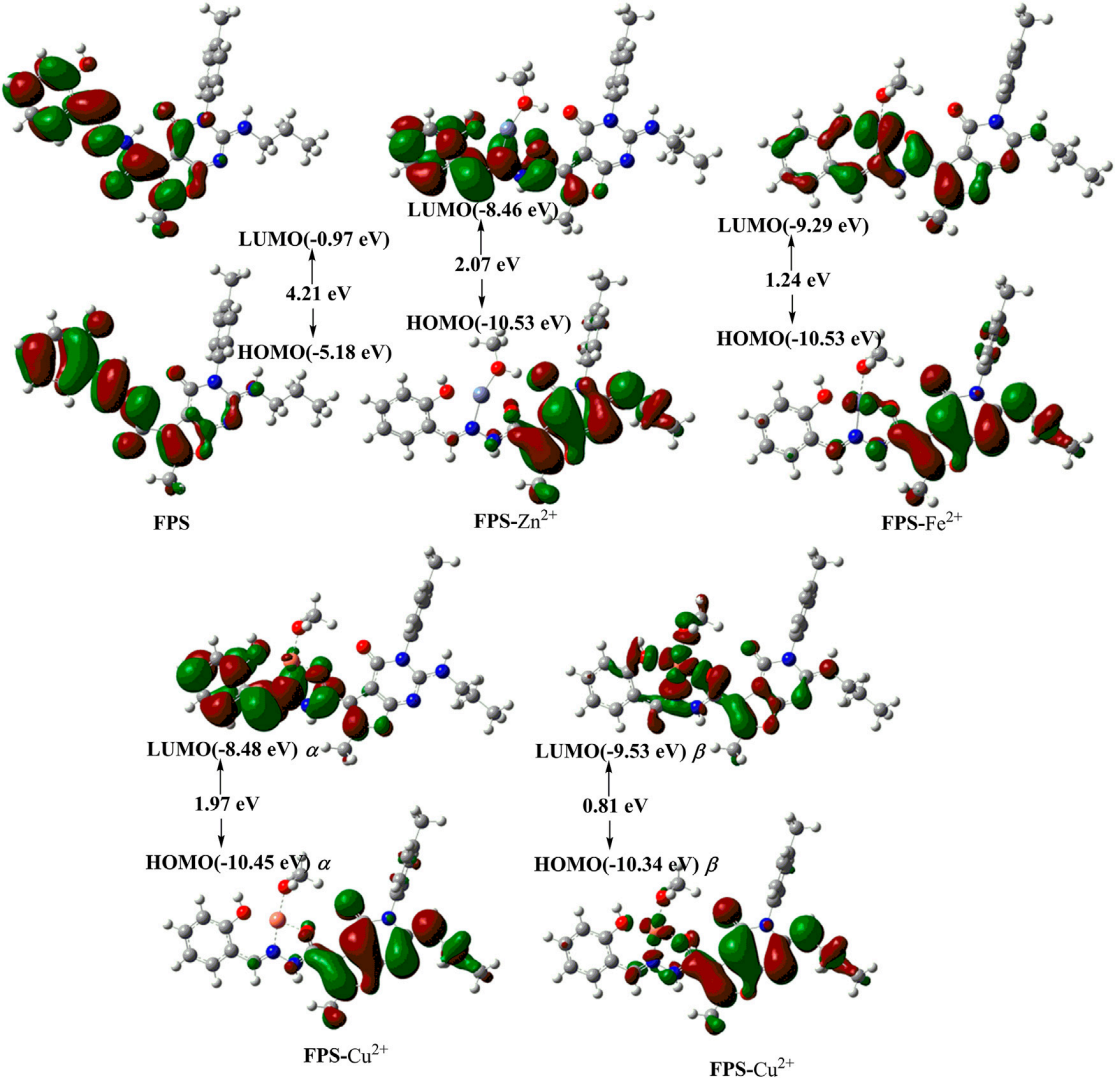
The optimized structures and Frontier molecular orbitals of FPS and FPS-X (X = Zn^2+^, Fe^2+^, Cu^2+^).

Based on the experimental data and Job’s plot, to further elucidate the influence of the structure on the electronic properties, DFT calculations are performed for **FPS**, **FPS**-X (X = Zn^2+^, Fe^2+^, Cu^2+^). As shown in Figure 7, for complex **FPS**-X (X = Zn^2+^, Fe^2+^, Cu^2+^), the deprotonated O atom of phenolic hydroxyl, O atoms of carbonyl, imine and the O atom of methanol were coordinated with metal ions. The calculated distributions of molecular orbitals (HOMO, highest occupied molecular orbital; SOMO, single electron occupied molecular orbital; and LUMO, lowest unoccupied molecular orbital) are shown in Figure 7. The HOMO and LUMO of **FPS** are predominately determined by the phenol moiety, the bride including acyl hydrazine moiety, furan moiety, and sectionally listed in pyridine moiety, respectively. Once **FPS** coordinated to metal ions (Zn^2+^, Fe^2+^, Cu^2+^), the HOMO/SOMO and LUMO in **FPS**-X (X = Zn^2+^, Fe^2+^, Cu^2+^) were conversely localized on the pyridine moiety and metal ions one, respectively. Apparently, this phenomenon was attributed to the enlargement of the conjugated system due to the complexation of the **FPS** and metal ions. The lower energy gap between the HOMO and LUMO level of **FPS**-X (X = Zn^2+^, Fe^2+^, Cu^2+^) compared with 4.21 eV of **FPS** was in good agreement with the red shift of the experimental fluorescence spectra (Figure 2B). Furthermore, the calculational binding energy *E*_bind_ (Supplementary Table S1) of **FPS**-X (X = Zn^2+^,Fe^2+^, Cu^2+^) show that the minimal values of **FPS**-Zn^2+^ and the maximum values of **FPS**-Cu^2+^ which cause the greater complexation of Cu^2+^compared with Zn^2+^ and Fe^2+^, probably further leading to the fluorescence quenching of **FPS**-Cu^2+^.

Fluorescence calculations of **FPS**-X (X = Zn^2+^, Fe^2+^, Cu^2+^) (Supplementary Tables S2–S4) was in good agreement with the experimental fluorescence data.

### Fluorescence Imaging in Living Cells

**FIGURE 8:**
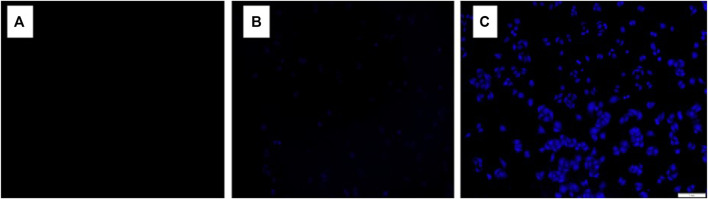
CLSM images of Hela cells treated under various conditions: **(A)** Hela cells were incubated with culture medium as a control; **(B)** Hela cells were cultured with probe FPS solution (5.0 μmol/L) for 25 min; **(C)** Hela cells were cultured with probe FPS solution (5.0 μmol/L) for 25 min, and after being washed with PBS and further incubated with Zn^2+^ (10.0 μmol/L) were then incubated for 5 min.

Hela cells were cultured in DMEM medium containing 10% fetal bovine serum cell culture medium with 5% CO_2_ atmosphere at 37°C. The cells were transferred into 24-well plates and incubated for 24 h at 37°C. The first group Hela cells treated in a culture medium (DMSO: DMEM = 1: 99, v/v) alone were used as a control (Figure 8A). In group 2 and 3, Hela cells were cultured with probe **FPS** solution (5.0 μmol/L) for 25 min (Figure 8B). In groups 4 and 5, Hela cells were cultivated successively with probe **FPS** (5.0 μmol/L and 10.0 μmol/L) for 25 min, after being washed with PBS, and further incubated with Zn^2+^ (10.0 μmol/L and 20.0 μmol/L) for 5 min, respectively (Figure 8C).

## Conclusion

This work was to synthesize and discover a specific “Dual-Response” to Zn^2+^ and Cu^2+^ probe based on a Schiff base bearing furopyrimidinone scaffold. The probe **FPS** with excellent linear relationship for the Zn^2+^ detection showed good and weak potential in imaging the exogenous and endogenous Zn^2+^, respectively, which can lead to the exploitation of growingly specific probes, particularly fluorescent for detection of Zn^2+^, Cu^2+^ and diagnosis of Zn^2+^, Cu^2+^ related diseases. In addition, the DFT calculations results showed how the structure affects the fluorescent behavior of **FPS**, which may help us to understand the essence of metal ions regulating effect in nature, and even give valuable reference to extend the real application of cell imaging (Li et al., 2021), imaging-guided (Sun et al., 2019), stimuli-responsive bioimaging (Min et al., 2021).

## Data Availability

The original contributions presented in the study are included in the article/Supplementary Material, further inquiries can be directed to the corresponding authors.

## References

[B1] Bae J.-E. Kim I. J. Nam K. H. (2018). Spectroscopic Analysis of the Cu2+-Induced Fluorescence Quenching of Fluorescent Proteins Amcyan and Morange2. Mol. Biotechnol. 60, 485–491. 10.1007/s12033-018-0088-1 29785699

[B2] Chae J. B. Lee H. Kim C. (2020). Determination of Zinc Ion by a Quinoline-Based Fluorescence Chemosensor. J. Fluoresc. 30, 347–356. 10.1007/s10895-020-02501-6 32040795

[B3] Domingo J. L. (1994). Metal‐induced Developmental Toxicity in Mammals: A Review. J. Toxicol. Environ. Health 42, 123–141. 10.1080/15287399409531868 8207750

[B4] Gao H.-T. Wang H.-M. Hou N. Guo X.-R. Zeng X.-H. Hu Y.-G. (2019). Synthesis, Crystal Structure and Antitumor Activities of 2-Acyl-Beta-Lactam-2-Carboxamides. Chin. J. Struct. Chem. 38, 416–421.

[B5] Garza-Lombó C. Posadas Y. Quintanar L. Gonsebatt M. E. Franco R. (2018). Neurotoxicity Linked to Dysfunctional Metal Ion Homeostasis and Xenobiotic Metal Exposure: Redox Signaling and Oxidative Stress. Antioxid. Redox Signaling 28, 1669–1703. 10.1089/ars.2017.7272 PMC5962337 29402131

[B6] Hershfinkel M. (2018). The Zinc Sensing Receptor, Znr/Gpr39, in Health and Disease. Ijms 19, 439. 10.3390/ijms19020439 PMC5855661 29389900

[B7] Hildebrand M. S. Phillips A. M. Mullen S. A. Adlard P. A. Hardies K. Damiano J. A. (2015). Loss of Synaptic Zn2+ Transporter Function Increases Risk of Febrile Seizures. Sci. Rep. 5, 17816. 10.1038/srep17816 26647834 PMC4673435

[B8] Hou N. Man J. H. Wang X. Y. He S. J. Li Q. Hu Y. G. (2019). Efficient Synthesis and Biological Evaluation of 2,4‐Diaminothieno[2,3‐ D ]pyrimidine Derivative. ChemistrySelect 4, 4901–4904. 10.1002/slct.201900123

[B9] Hu Y.-G. Wang Y. Du S.-M. Chen X.-B. Ding M.-W. (2010). Efficient Synthesis and Biological Evaluation of Some 2,4-Diamino-Furo[2,3-D]pyrimidine Derivatives. Bioorg. Med. Chem. Lett. 20, 6188–6190. 10.1016/j.bmcl.2010.08.122 20850310

[B10] Hu Y.-G. Zheng A.-H. Li G.-J. Dong M.-Z. Ye F. Sun F. (2014). Efficient Synthesis of New Thieno 2,3-D Pyrimidin-4(3h)-One Derivatives for Evaluation as Anticancer Agents. J. Heterocycl. Chem. 51, 84–88. 10.1002/jhet.1823

[B11] Hu Y. Gao H. Wang G. Wang Y. Qu Y. Xu J. (2012). Synthesis and Antitumor Activity of Some 2-Amino-Furo[2,3-*D*]- Yrimidin-4(3*h*)-One Derivatives. Chin. J. Org. Chem. 32, 1468–1472. 10.6023/cjoc201203003

[B12] Isaev N. K. Stelmashook E. V. Genrikhs E. E. (2020). Role of Zinc and Copper Ions in the Pathogenetic Mechanisms of Traumatic Brain Injury and Alzheimer's Disease. Rev. Neurosci. 31, 233–243. 10.1515/revneuro-2019-0052 31747384

[B13] Kim H. Sarkar S. Nandy M. Ahn K. H. (2021). Imidazolyl-Benzocoumarins as Ratiometric Fluorescence Probes for Biologically Extreme Acidity. Spectrochimica Acta A: Mol. Biomol. Spectrosc. 248, 119088. 10.1016/j.saa.2020.119088 33187882

[B14] Klenner M. A. Pascali G. Massi M. Fraser B. H. (2021). Fluorine‐18 Radiolabelling and Photophysical Characteristics of Multimodal PET-Fluorescence Molecular Probes. Chem. Eur. J. 27, 861–876. 10.1002/chem.202001402 32697376

[B15] Li Q. Chen Y.-M. Hu Y.-G. Luo X. Ko J. K. S. Cheung C. W. (2016). Synthesis and Biological Activity of Fused Furo[2,3-D]pyrimidinone Derivatives as Analgesic and Antitumor Agents. Res. Chem. Intermed. 42, 939–949. 10.1007/s11164-015-2064-8

[B16] Li R.-H. Feng X.-Y. Zhou J. Yi F. Zhou Z.-Q. Men D. (2021). Rhomboidal Pt(Ii) Metallacycle-Based Hybrid Viral Nanoparticles for Cell Imaging. Inorg. Chem. 60, 431–437. 10.1021/acs.inorgchem.0c03095 33320662

[B17] Liang L. Lan F. Ge S. Yu J. Ren N. Yan M. (2017). Metal-Enhanced Ratiometric Fluorescence/Naked Eye Bimodal Biosensor for Lead Ions Analysis with Bifunctional Nanocomposite Probes. Anal. Chem. 89, 3597–3605. 10.1021/acs.analchem.6b04978 28235180

[B18] Liu J. Xie Y. Yang Q. Huang N. Wang L. (2021). Ugi Four-Component Reaction Based on the *In Situ* Capture of Amines and Subsequent Modification Tandem Cyclization Reaction: "One-Pot" Synthesis of Six- and Seven-Membered Heterocycles. Chin. J. Org. Chem. 41, 2374–2383. 10.6023/cjoc202012040

[B19] Liu M.-G. Liu N. Xu W.-H. Wang L. (2019a). Tandem Reaction Strategy of the Passerini/Wittig Reaction Based on the *In Situ* Capture of Isocyanides: One-Pot Synthesis of Heterocycles. Tetrahedron 75, 2748–2754. 10.1016/j.tet.2019.03.057

[B20] Liu N. Chao F. Liu M.-G. Huang N.-Y. Zou K. Wang L. (2019b). Odorless Isocyanide Chemistry: One-Pot Synthesis of Heterocycles via the Passerini and Postmodification Tandem Reaction Based on the *In Situ* Capture of Isocyanides. J. Org. Chem. 84, 2366–2371. 10.1021/acs.joc.8b03242 30676019

[B21] Ma L. Wu G. Li Y. Qin P. Meng L. Liu H. (2015). A Reversible Metal Ion Fueled DNA Three-Way Junction Molecular Device for "Turn-On and -Off" Fluorescence Detection of Mercury Ions (Ii) and Biothiols Respectively with High Selectivity and Sensitivity. Nanoscale 7, 18044–18048. 10.1039/c5nr04688b 26487480

[B22] Marchetti C. (2014). Interaction of Metal Ions with Neurotransmitter Receptors and Potential Role in Neurodiseases. Biometals 27, 1097–1113. 10.1007/s10534-014-9791-y 25224737

[B23] Min X. Zhang J. Li R.-H. Xia F. Cheng S.-Q. Li M. (2021). Encapsulation of Nir-Ii Aiegens in Virus-like Particles for Bioimaging. ACS Appl. Mater. Inter. 13, 17372–17379. 10.1021/acsami.1c02691 33834757

[B24] Nsanzamahoro S. Cheng W. Mutuyimana F. P. Li L. Wang W. Ren C. (2020). Target Triggered Fluorescence "Turn-Off" of Silicon Nanoparticles for Cobalt Detection and Cell Imaging with High Sensitivity and Selectivity. Talanta 210, 120636. 10.1016/j.talanta.2019.120636 31987169

[B25] Park J.-S. Blair N. F. Sue C. M. (2015). The Role of Atp13a2 in Parkinson's Disease: Clinical Phenotypes and Molecular Mechanisms. Mov Disord. 30, 770–779. 10.1002/mds.26243 25900096

[B26] Sannigrahi A. Chowdhury S. Das B. Banerjee A. Halder A. Kumar A. (2021). The Metal Cofactor Zinc and Interacting Membranes Modulate Sod1 Conformation-Aggregation Landscape in an *In Vitro* Als Model. Elife 10, 61453. 10.7554/elife.61453 PMC8087447 33825682

[B27] Schmidt K. Wolfe D. M. Stiller B. Pearce D. A. (2009). Cd2+, Mn2+, Ni2+ and Se2+ Toxicity to *Saccharomyces cerevisiae* Lacking YPK9p the Orthologue of Human ATP13A2. Biochem. Biophysical Res. Commun. 383, 198–202. 10.1016/j.bbrc.2009.03.151 PMC3906623 19345671

[B28] Sun Y. Ding F. Chen Z. Zhang R. Li C. Xu Y. (2019). Melanin-Dot-Mediated Delivery of Metallacycle for Nir-Ii/Photoacoustic Dual-Modal Imaging-Guided Chemo-Photothermal Synergistic Therapy. Proc. Natl. Acad. Sci. USA. 116, 16729–16735. 10.1073/pnas.1908761116 31391305 PMC6708342

[B29] Wang C. Zhang R. Wei X. Lv M. Jiang Z. (2020). “Metalloimmunology: The Metal Ion-Controlled Immunity,” in Advances in Immunology in China, Pt B. Editors Dong C. Jiang Z., 187–241. 10.1016/bs.ai.2019.11.007 32081198

[B30] Wang L. Frei M. S. Salim A. Johnsson K. (2019). Small-Molecule Fluorescent Probes for Live-Cell Super-resolution Microscopy. J. Am. Chem. Soc. 141, 2770–2781. 10.1021/jacs.8b11134 30550714

[B31] Wang L. Guan Z.-R. Ding M.-W. (2016a). One-Pot Synthesis of 1h-Isochromenes and 1,2-Dihydroisoquinolines by a Sequential Isocyanide-Based Multicomponent/Wittig Reaction. Org. Biomol. Chem. 14, 2413–2420. 10.1039/c5ob02405f 26810599

[B32] Wang L. Xie Y.-B. Huang N.-Y. Yan J.-Y. Hu W.-M. Liu M.-G. (2016b). Catalytic Aza-Wittig Reaction of Acid Anhydride for the Synthesis of 4H-Benzo[d][1,3]oxazin-4-Ones and 4-Benzylidene-2-Aryloxazol-5(4h)-Ones. ACS Catal. 6, 4010–4016. 10.1021/acscatal.6b00165

[B33] Yang H. Wu Y. Tian F. (2019). A Fluorescent Sensor for Cu2+ Ion with High Selectivity and Sensitivity Based on Ict and Pet. J. Fluoresc. 29, 1153–1159. 10.1007/s10895-019-02406-z 31420781

[B34] Yin H. Z. Wang H.-L. Ji S. G. Medvedeva Y. V. Tian G. Bazrafkan A. K. (2019). Rapid Intramitochondrial Zn2+ Accumulation in Ca1 Hippocampal Pyramidal Neurons after Transient Global Ischemia: A Possible Contributor to Mitochondrial Disruption and Cell Death. J. Neuropathol. Exp. Neurol. 78, 655–664. 10.1093/jnen/nlz042 31150090 PMC6581555

