# Supplementary material for: Synthesis and Discovery of Schiff Base Bearing Furopyrimidinone for Selective Recognition of Zn2+ and its Applications in Cell Imaging and Detection of Cu2+
Source: Front Chem. 2021 Nov 29;9:774090. doi: 10.3389/fchem.2021.774090 (PMC8666604; doi:10.3389/fchem.2021.774090)
Supplement: Supplementary file 1 [file DataSheet1.docx]

***Supplementary Material***

**Theoretical calculations**

The ground-state geometric optimizations and fluorescence calculations were performed with the Gaussian16 program package based on the density functional theory (DFT)/time-dependent density functional theory (TDDFT) method^[1]^. Becke's threeparameter exchange functional^[2,3]^ along with the Lee–Yang–Parr correlation functional with the restricted (B3LYP) was employed for all the calculations. The standard split valence plus polarization function 6-31G* basis set was used to treat all atoms. The fully optimized stationary points were further analyzed by harmonic vibrational frequency to ensure that real local minima had been found without imaginary vibrational frequency. And the solvent model density (SMD) were used to consider the solvation effect of methanol.

**Table S1**. The binding energy: X + **FPS** + methanol **→ FPS**-X (X = Zn^2+^, Fe^2+^, Cu^2+^)

| X | ΔE_bind_(kcal/mol) |
| --- | --- |
| Zn^2+^ | -378.79 |
| Fe^2+^ | -387.02 |
| Cu^2+^ | -397.04 |

**Table S2.** Calculated electronic transition data in terms of excitation energies (E/eV), emission wavelengths (λ_em_/nm), oscillator strengths (*f*_em_), and electronic compositions for **FPS-**Zn^2+^ in CH_3_OH using TD-B3LYP/6-31G* on the SMD model.

| Electronic transition^a^ | Excitation energy (λ_em_/nm) | f ^b^ | Composition ^c^ | CI ^d^ |
| --- | --- | --- | --- | --- |
| S_0_→S_1_  S_0_→S_2_  S_0_→S_3_ | 2.19 eV (566 nm)  3.46 eV (359 nm)  3.76 eV (329 nm) | 0.0833  0.6336  0.2138 | H→L  H-1→L  H-4→L | 0.99  0.91  0.38 |
| S_0_→S_3_ | 3.76 eV (329 nm) | 0.2138 | H→L+1 | 0.49 |
| S_0_→S_4_ | 3.80 eV (326 nm) | 0.1410 | H-2→L | 0.47 |
| S_0_→S_5_ | 3.84 eV (323 nm) | 0.3022 | H-4→L+1 | 0.45 |

^a^ Only selected excitation states were considered. The numbers in parentheses are the excitation energy in wavelength. ^b^ Oscillator strength. ^c^ H stands for HOMO and L stands for LUMO. Only the configurations are presented. ^d^ Coefficient of the wave function for excitations. The CI coefficients are in absolute values.

**Table S3.** Calculated electronic transition data in terms of excitation energies (E/eV), emission wavelengths (λ_em_/nm), oscillator strengths (*f*_em_), and electronic compositions for **FPS-**Fe^2+^ in CH_3_OH using TD-B3LYP/6-31G* on the SMD model.

| Electronic transition^a^ | Excitation energy (λ_em_/nm) | f ^b^ | Composition ^c^ | CI ^d^ |
| --- | --- | --- | --- | --- |
| S_0_→S_1_  S_0_→S_2_  S_0_→S_3_ | -0.27 eV (-4569 nm)  0.57 eV (2161 nm)  0.61 eV (2040 nm) | 0.0000  0.0002  0.0000 | H-2→L  H-1→L  H-6→L | 0.69  0.57  0.56 |
| S_0_→S_4_ | 2.17 eV (571 nm) | 0.0006 | H-1→L+3 | 0.66 |
| S_0_→S_5_ | 2.24 eV (554 nm) | 0.1115 | H→L | 0.68 |

^a^ Only selected excitation states were considered. The numbers in parentheses are the excitation energy in wavelength. ^b^ Oscillator strength. ^c^ H stands for HOMO and L stands for LUMO. Only the configurations are presented. ^d^ Coefficient of the wave function for excitations. The CI coefficients are in absolute values.

**Table S4.** Calculated electronic transition data in terms of excitation energies (E/eV), emission wavelengths (λ_em_/nm), oscillator strengths (*f*_em_), and electronic compositions for **FPS-**Cu^2+^ in CH_3_OH using TD-B3LYP/6-31G* on the SMD model.

| Electronic transition^a^ | Excitation energy (λ_em_/nm) | f ^b^ | Composition ^c^ | CI ^d^ |
| --- | --- | --- | --- | --- |
| S_0_→S_1_  S_0_→S_2_  S_0_→S_3_ | 0.55 eV (2242 nm)  0.69 eV (1800 nm)  0.73 eV (1687 nm) | 0.0003  0.0002  0.0002 | H-11→L  H-14→L  H-12→L | 1.03  0.79  0.89 |
| S_0_→S_4_ | 1.02 eV (1209 nm) | 0.0006 | H-24→L | 0.42 |
| S_0_→S_5_ | 1.34 eV (924 nm) | 0.0060 | H→L | 1.98 |

^a^ Only selected excitation states were considered. The numbers in parentheses are the excitation energy in wavelength. ^b^ Oscillator strength. ^c^ H stands for HOMO and L stands for LUMO. Only the configurations are presented. ^d^ Coefficient of the wave function for excitations. The CI coefficients are in absolute values.

**References**

[1] M. J. Frisch, G. W. Trucks, H. B. Schlegel, G. E. Scuseria, M. A. Robb, J. R. Cheeseman, G. Scalmani, V. Barone, B. Mennucci, G. A. Petersson, H. Nakatsuji, M. Caricato, X. Li, H. P. Hratchian, A. F. Izmaylov, J. Bloino, G. Zheng, J. L. Sonnenberg, M. Hada, M. Ehara, K. Toyota, R. Fukuda, J. Hasegawa, M. Ishida, T. Nakajima, Y. Honda, O. Kitao, H. Nakai, T. Vreven, J. A. Montgomery, J. E. Peralta Jr, F. Ogliaro, M. Bearpark, J. J. Heyd, E. Brothers, K. N. Kudin, V. N. Staroverov, R. Kobayashi, J. Normand, K. Raghavachari, A. Rendell, J. C. Burant, S. S. Iyengar, J. Tomasi, M. Cossi, N. Rega, J. M. Millam, M. Klene, J. E. Knox, J. B. Cross, V. Bakken, C. Adamo, J. Jaramillo, R. Gomperts, R. E. Stratmann, O. Yazyev, A. J. Austin, R. Cammi, C. Pomelli, J. W. Ochterski, R. L. Martin, K. Morokuma, V. G. Zakrzewski, G. A. Voth, P. Salvador, J. J. Dannenberg, S. Dapprich, A. D. Daniels, O. Farkas, J. B. Foresman, J. V. Ortiz, J. Cioslowski and D. J. Fox, Gaussian 16, Revision C.01; Gaussian Inc., Wallingford, CT, 2016.

[2] A. D. Becke, J. Chem. Phys., 1993, 98, 5648–5652.

[3] W. Wang, Q. Wen, Y. Zhang, X. L. Fei, Y. X. Li, Q. B. Yang, X. Y. Xu, Dalton Trans., 2013, 42, 1827–1833.
